# Preparation of Poly (Allylthiourea-Co-Acrylic Acid) Derived Carbon Materials and Their Applications in Wastewater Treatment

**DOI:** 10.3390/molecules24050957

**Published:** 2019-03-08

**Authors:** Limei Liang, Chengpeng Li, Tingting Hou, Zhiying Zhong, Dongchu Chen, Sidong Li, Zhang Hu, Haihua Yang, Xiufang Ye

**Affiliations:** 1 School of Materials Science and Energy Engineering, Foshan University, Foshan 528000, China; llmgdou2017@163.com (L.L.); lcp0802@126.com (C.L.); chen@fosu.edu.cn (D.C.); 2 Faculty of Chemistry and Environmental Science, Guangdong Ocean University, Zhanjiang 524088, China; htt0415@126.com (T.H.); zzy15767018762@163.com (Z.Z.); lisidong2210491@163.com (S.L.); huzhcarrot@163.com (Z.H.); gdouyanghaihua@163.com (H.Y.)

**Keywords:** poly allylthiourea, poly acrylic acid, carbonization, adsorption, wastewater treatment

## Abstract

Functional carbon materials have been developed and applied in various sewage treatment applications in recent years. This article reports the fabrication, characterization, and application of a new kind of poly (allylthiourea-co-acrylic acid) (PAT–PAC) hydrogel-based carbon monolith. The results indicated that the poly acrylic acid component can endow the PAT–PAC hydrogel with an increased swelling ratio and enhanced thermal stability. During the carbonization process, O–H, N–H, C=N, and –COO– groups, etc. were found to be partly decomposed, leading to the conjugated C=C double bonds produced and the clear red shift of C=O bonds. Particularly, it was found that this shift was accelerated under higher carbonization temperature, which ultimately resulted in the complex conjugated C=C network with oxygen, nitrogen, and sulfur atoms doped in-situ. The as-obtained carbon monoliths showed good removal capacity for Ni(II) ions, organic solvents, and dyes, respectively. Further analysis indicated that the Ni(II) ion adsorption process could be well described by pseudo-second-order and Freundlich models under our experimental conditions, respectively. The adsorption capacity for Ni(II) ions and paraffin oil was as high as 557 mg/g and 1.75 g/g, respectively. More importantly, the as-obtained carbon monoliths can be recycled and reused for Ni(II) ions, acetone, and paraffin oil removal. In conclusion, the proposed PAT–PAC-based carbonaceous monoliths are superior adsorbents for wastewater treatment.

## 1. Introduction

In the last several decades, both accelerated industrialization and urbanization, producing a great deal of effluent with heavy metal ions, organic solvents, and dyes, has posed a great challenge to human health and ecological balance [1]. Those pollutants are extremely toxic to the ecosystem even at very low concentrations [2]. Hence, eradication of those harmful wastes is extremely important for environmental protection. Various methods have been developed to remove heavy metal ions, organic solvents, and dyes from industrial wastewaters, including ion exchange [3], precipitation, adsorption, membrane separation [4], filtration [5], sedimentation, chemical oxidation, and so on. Of these methods above, adsorption has been found to be an efficient way to achieve effective separation due to its economic viability, simplicity, and high efficiency [6]. 

Allylthiourea, a monomer with both nitrogen and sulfur atoms, is a good ligand and can form stable complexes with various ions, including Zn^2+^, Cd^2+^, Hg^2+^, Cu^2+^, and Au^3+^, etc. [7,8]. Thus, a couple of allylthiourea based homopolymer or copolymer adsorbents have been developed for heavy metal-contaminated effluent treatment [8,9,10,11]. For instance, Zhang and co-author [12] synthesized magnetic poly allylthiourea nanoparticles and found that the adsorption capacity of the as-synthesized particles for Hg^2+^ ions was as high as 78.3 mg g^−1^. In pursuit of effective chelation, other functional groups were also introduced to produce the new active sites in poly allylthiourea-based adsorbents [10,11,12]. Recently, it was found that both C=O (in poly acrylic acid) and C=S (in poly allylthiourea) functional groups in chitosan-graft-poly (allylthiourea-co-acrylic acid) granular hydrogels can chelate with Ni^2+^ ions effectively [12], showing that poly-(allylthiourea-co-acrylic acid)-based adsorbents are superior candidates for industrial sewage treatment.

Apart from the polymer adsorbents discussed above, carbon-based materials including activated carbon and carbon black have been widely applied in practical sewage treatment for decades [13]. Particularly, recent research indicated that porous carbon materials possess superior adsorption properties for heavy metal ions [14,15], organic solvents (or oils) [15,16,17], and toxic dyes [18,19], due to their high surface area [14,19], Normally, porous carbon materials were fabricated via direct pyrolysis of synthetic polymer materials (i.e., imine-linked polymers [20], resorcinol–formaldehyde resin [21], and poly (benzoxazine–coresol) [19]), or natural polymeric compounds (i.e., bamboo leaves [22] and pomelo peel [19]). Considering the superior properties of the poly-(allylthiourea-co-acrylic acid)-based materials mentioned above, it is anticipated that poly-(allylthiourea-co-acrylic acid)-based porous carbon material may combine the advantages of both poly-(allylthiourea-co-acrylic acid) and porous carbon materials. However, to our best knowledge, there have been no explorations or reports of poly-(allylthiourea-co-acrylic acid) based carbonaceous materials so far.

Based on the research background presented above, this work aimed at fabricating a new kind of porous carbon monolith using poly (allylthiourea-co-acrylic acid) hydrogels as precursors. The chemical structure, carbonization yield, and surface morphology of the as-fabricated porous adsorbents were systematically discussed. In addition, the adsorption ability of the adsorbents for various simulated contaminants in wastewater, such as heavy metal ions, organic solvents, and dyes, was also systematically discussed. Our results show that the porous carbon monoliths can serve as versatile adsorbents for wastewater treatment.

## 2. Results

### 2.1. Characterizations

During the synthesis process, we found that the crosslinked poly allylthiourea (PAT) hydrogel was very fragile. However, the poly acrylic acid (PAC) component introduced could greatly improve its ductility and dimensional stability. Thus, poly (allylthiourea-co-acrylic acid) (PAT–PAC) hydrogels could be used for carbonaceous monolith fabrication. Preliminary investigation (Figure S1) indicated that the swelling ratio of PAT–PAC hydrogels increased quickly at the initial stage and reached equilibrium around 6–8 h. The equilibrium swelling ratio of hydrogel was affected greatly by the allylthiourea (AT)/acrylic acid (AA) ratio and pH values. To elaborate further, PAT–PAC-70 had the best ability to hold water, followed by PAT–PAC-50, and PAT–PAC-30. AA contained the hydrophilic groups –COOH, which could form hydrogen bonds with water molecules or ionize in a basic environment, while AT units were hydrophobic [23]. Thus, it can be deduced that PAT–PAC hydrogels were synthesized successfully. 

**Figure 1:**
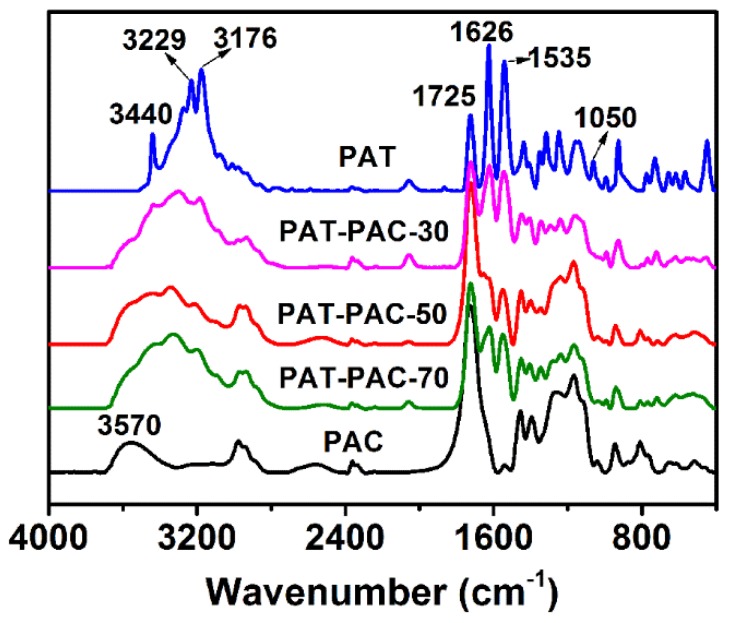
FTIR of dry hydrogels. PAT is poly allylthiourea, PAC is poly acrylic acid, PAT–PAC is poly (allylthiourea-co-acrylic acid).

FTIR was then used to analyze the molecular structure of the PAT–PAC-n synthesized. As can be seen from Figure 1, the characteristic peaks centered at 3440 cm^−1^, 1725 cm^−1^, 1626 cm^−1^, and 1050 cm^−1^ were assigned to the absorptions of the –NH_2_, C=O, C=N, and C=S bands in the crosslinked PAT, respectively [7,8]. For PAC, the broad band between 3340 and 3683 cm^−1^ corresponded to the stretching absorption of –OH, and the strong absorption peak located at 1725 cm^−1^ was attributed to the stretching vibration of C=O [19]. Compared to the pure PAT and PAC, the copolymers PAT–PAC-30, PAT–PAC-50, and PAT–PAC-70 contained all the characteristic peaks discussed above, indicating that the poly (allylthiourea-co-acryl acids) were successfully polymerized and crosslinked. Thus, the three-dimensional networks with abundant thiourea and carboxyl groups were successfully constructed, which could potentially form effective coordination linkages with heavy metal ions.

**Figure 2:**
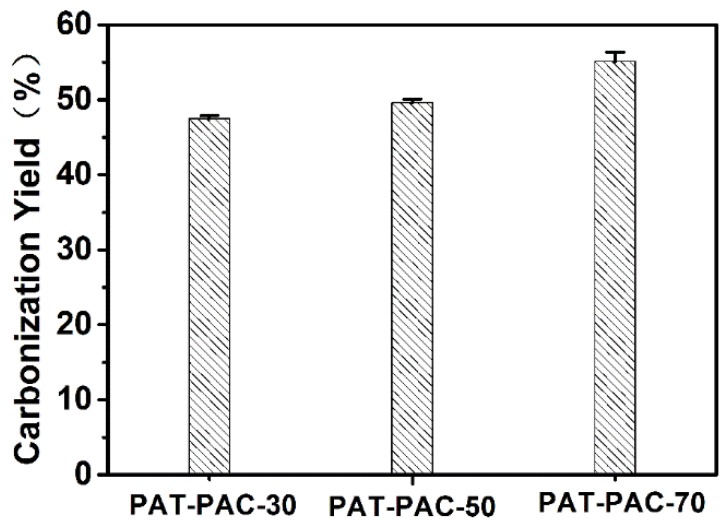
Carbonization yield of PAT–PAC hydrogels carbonized at 300 °C.

According to the thermal analysis (Figure S2), there were four stages involved in the PAT–PAC-n decomposition process. The first weight loss within the temperature range 100–170 °C could be due to dehydration. The weight loss aggravated between 180 and 253 °C, which could be the onset of decomposition of the polymer unit. After this stage, both PAT–PAC-50 and PAT–PAC-30 were degraded at 254–378 °C and PAT–PAC-70 at 254–354 °C. The last step, which started at 379 or 355 °C, was ascribed to the thermal decomposition of allylthiourea, acryl acid in the polymer networks, and other side groups of copolymer. At 600 °C, PAT–PAC-30 had the highest residual weight percentage of 13.04%, followed by PAT–PAC-50 (11.34%), and PAT–PAC-70 (9.70%). Since the residual weight percentage of the as-synthesized PAT–PAC-n hydrogels at 600 °C was extremely low, we used the relative low temperature (300 °C) for PAT–PAC-n hydrogel carbonization (Figure 2). The carbonization yields of all hydrogels were over 40%; the PAT–PAC-70 had the highest carbonization yield of 55.13%, followed by PAT–PAC-50 (49.61%), and PAT–PAC-30 (47.51%), indicating that the thermal stability of AT was poorer than AA. Furthermore, this trend was basically consistent with the thermal analysis shown in Figure S2.

**Figure 3:**
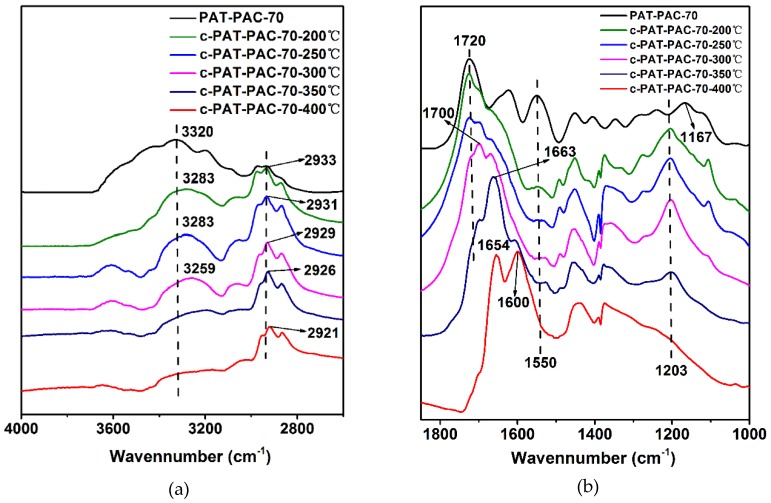
FTIR of the chemical structure changes during the carbonization: (**a**) the spectra within the spectral ranges of 4000–2600 cm^−1^ and (**b**) the spectra within the spectral ranges of 1850–1000 cm^−1^.

To explore the carbonization mechanism, the chemical structure changes during carbonization were analyzed using FTIR; as seen in Figure 3, all the spectra of carbon monoliths showed great differences compared to the original PAT–PAC-70. The wide peak centered at 3320 cm^−1^ was assigned to O–H and N–H [24], and the triplet bands at 3000–2800 cm^−1^ belonged to the C–H stretching vibration [25,26] (Figure 3a). Both the peaks of O–H and N–H shifted to low wavenumbers after carbonization. Particularly, those two peaks were much weaker after carbonization at relative low carbonization temperature (200, 250, and 300 °C), but nearly invisible at high carbonization temperature (350 and 400 °C), suggesting the possible degradation of the groups (O–H and N–H) during the carbonization. It was also found that the triplet bands of C–H shifted to low wavenumbers, and this shift was accelerated under the higher carbonization temperature [27]. In addition, the characteristic bands around 1720, 1623, and 1550 cm^−1^ were ascribed to the C=O, C=N, and symmetric –COO– stretching vibration, respectively. After carbonization, the C=N signal was nearly invisible and the –COO– signal was much weaker. It was also found that the C=O vibration showed a gradually red shift to lower wavenumbers with the improvement of the carbonization temperature. Meanwhile, the shoulder peak located around 1700–1670 appeared after the carbonization (i.e., 200, 250, and 300 °C). This can be explained by the formation of the single and conjugated C=C double bonds next to the residual C=O groups during the pyrolysis [28]. When the temperature was set as 350 °C, the shoulder peak shifted to 1663 and dominated the absorbance. With further increases in the carbonization temperature to 400 °C, the C=O vibration was completely invisible and the C=C vibration split into two peaks at 1653 and 1600 cm^−1^, indicating that two different conjugated C=C systems may have been produced and the residual C=O groups were not detectable. Moreover, the peak of O–C=O at 1167 cm^−1^ and 1239 cm^−1^ [7,23] merged into a new single peak (1203 cm^−1^), which ultimately disappeared at the highest carbonization temperature of 400 °C. Therefore, it can be concluded that pyrolysis was accelerated under the higher carbonization temperature, leading to the loss of the functional groups (i.e., O–H, N–H, C=N, –COO–, and C=O, etc.) and the formation of the complex C=C conjugated network. However, relatively low carbonization temperature may have partly kept the functional groups, leading to the final carbon monoliths with multiple functions. Thus, 300 °C was ideal for carbonization.

**Figure 4:**
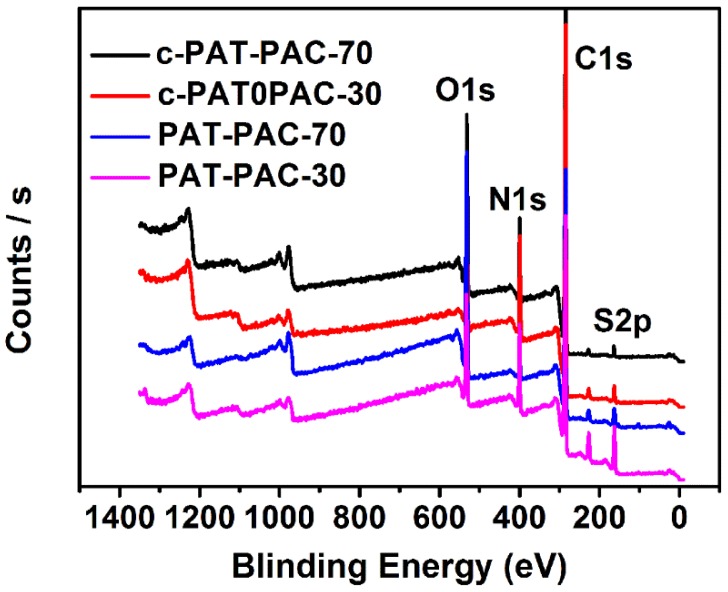
XPS spectra of the PAT–PAC and c-PAT–PAC.

X-ray photoelectron spectroscopy (XPS) was further used to analyze the elemental composition of PAT–PAC and c-PAT–PAC. As shown in Figure 4, both PAT–PAC and c-PAT–PAC samples contained C, O, S, and N elements. As expected, C contents were improved while O contents were clearly decreased after carbonization (Table 1). Meanwhile, S and N elements still remained after carbonization, and N contents were even slightly improved (10.07% for c-PAT–PAC-70 and 14.63% for c-PAT–PAC-30). The element composition changes indicated that C/N elements were more stable than O/S elements during the high temperature treatment. Due to the multiple elements that existed, the as-obtained carbon monoliths may have contained abundant functional groups and could potentially have formed chelates with metal ions.

**Figure 5:**
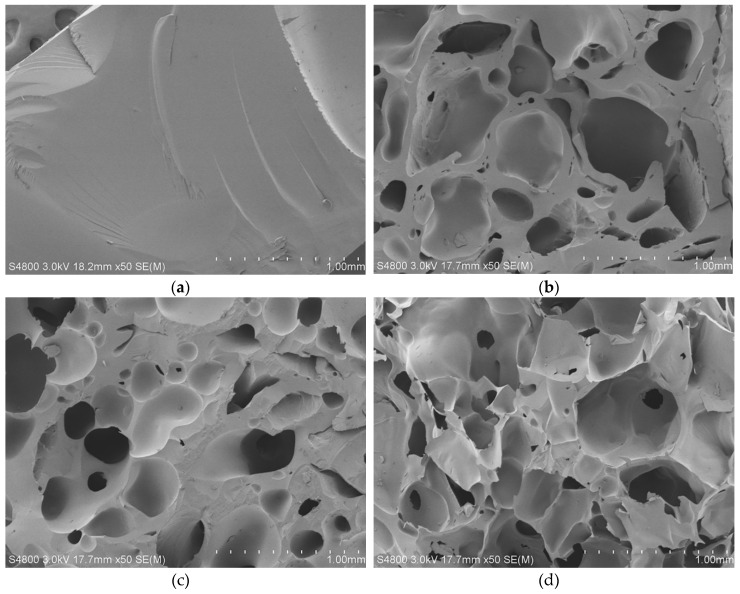
SEM images of (**a**) PAT–PAC-50, (**b**) c-PAT–PAC-70, (**c**) c-PAT–PAC-50, and (**d**) c-PAT–PAC-30.

Surface morphologies are shown in Figure 5. PAT–PAC-50 showed a smooth surface with no obvious pores (Figure 5a), whereas some pores existed on the surfaces of c-PAT–PAC-70, c-PAT–PAC-50, and c-PAT–PAC-30 (Figure 5b–d)). It was clear that the surface of carbon monoliths was coarse and undulant, increasing the surface area [29]. Morphology of carbon monoliths is important for its practical application. For pores in carbon monoliths, many small, interconnected pores were observed, and it was obvious that pore size in c-PAT–PAC-70 was about 0.25 mm while c-PAT–PAC-50 was 0.30 mm and c-PAT–PAC-30 was 0.35 mm; in summary, increasing AT resulted in a larger pore size. Therefore, the pore size of the carbon monoliths could be adjusted by controlling the molar amount of AT and AA. In addition, it was observed that the pore walls of carbon monoliths thinned with increasing AT, as a consequence of the PAT–PAC further swelling during the carbonization process [30]. These results indirectly reflect the thermal instability of AT, which is similar to Figure S2 and Figure 2.

### 2.2. Heavy Metal Ion Adsorption

**Figure 6:**
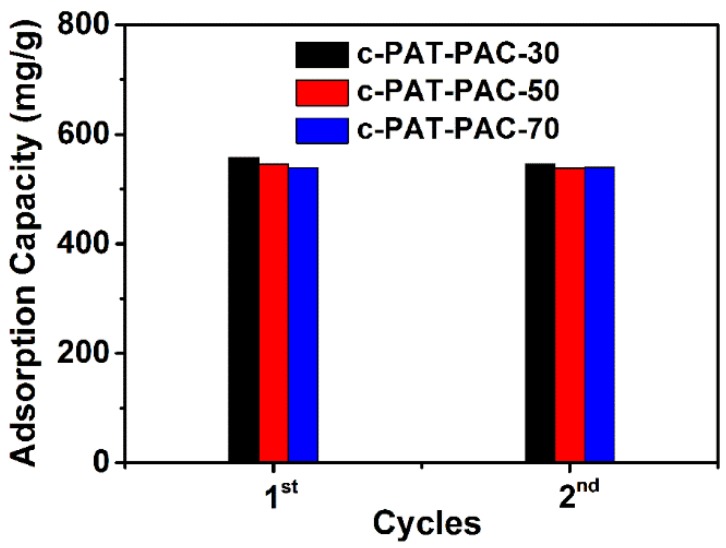
Ni(II) adsorption of PAT–PAC carbon monoliths.

Based on the Ni(II) adsorption (Figure 6), all carbon monoliths had a high Ni(II) adsorption more than 400 mg/L. Furthermore, with the addition of AT, the difference in adsorption capacity of Ni(II) was insignificant. It also can be seen that after two cycles, there was a slight decline or no decline for carbon monoliths. This, therefore, indicated the potential utility of the adsorbents on large scales. More importantly, PAT–PAC carbon monoliths had better Ni(II) adsorption capacity than other adsorbents in the literatures (Table 2), which indicates that PAT–PAC carbon monoliths are effective adsorbents to remove Ni(II) from wastewater.

In this study, the pseudo first-order model and pseudo second-order model were used to characterize the adsorption kinetics toward Ni(II), as expressed as follows [34,35]:

Pseudo first-order equation:

(1) $$log\left(q_{e}-q_{t}\right)=logq_{e}-\frac{K_{1}}{2.303}t,$$

Pseudo second-order equation:

(2) $$\frac{t}{q_{t}}=\frac{1}{K_{2}q_{e}{}^{2}}+\frac{t}{q_{e}},$$

where *q_e_* and *q_t_* are the amounts of Ni(II) adsorbed (mg/g) at equilibrium and at time *t*, respectively. *K_1_* is the adsorption rate constant of the pseudo-first-order model. *K_2_* is the overall rate constant of the pseudo-second-order adsorption (g/mg min), which can be determined from the straight-line plot of *t/q_t_* against *t*. The initial adsorption rate *h* (mg/g min) can be obtained when t approaches zero using the following equation [36]:

(3) $$h=k_{2}q_{e}{}^{2}.$$

**Figure 7:**
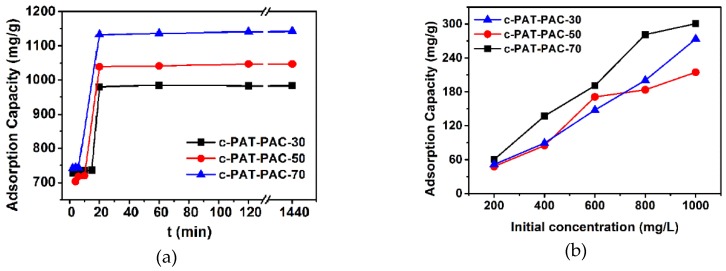
The plots of amount adsorbed for Ni(II) as a function of (**a**) contact time and (**b**) initial concentration.

The effect of contact time toward Ni(II) adsorption is shown in Figure 7a. The results indicated that the adsorption of Ni(II) onto porous carbon monoliths was very rapid and most of the Ni(II) could be adsorbed within 20 min. Since there was no detectable increase after 120 min, the adsorption saturation could be well realized within 120 min. The parameters of the pseudo-first-order model and pseudo-second-order model were calculated from slopes and intercepts of the lines (Figure S3). The characteristic parameters are summarized in Table 3. In terms of higher correlation coefficient values *R^2^* (*R^2^* > 0.99), the Ni(II) adsorption showed better compliance with the pseudo-second-order model. The initial adsorption rate of carbon monoliths was more than 400 mg/g min, this may be due to the availability of uncovered surface area [37,38]. 

In this part, adsorption of Ni(II) onto carbon monoliths was investigated at different initial concentrations. The plot of adsorption capacity against initial concentrations (Figure 7b) illustrated that adsorption capacity increased with the increasing initial concentration and the adsorption saturation was not achieved when the initial concentration reached 1000 mg/L. Due to the higher initial concentration, a larger driving force could be generated as a result of higher concentration gradient pressure, which could overcome the mass transfer resistance of Ni(II) ions from the aqueous phase to the solid phase, leading to a higher collision probability between Ni(II) ions and available adsorption sites [7]. In addition, three typical isotherms, including Langmuir, Freundlich, and Tempkin models, were used to fit the experimental data [39,40]:

Langmuir equation:

(4) $$\frac{C_{e}}{q_{e}}=\frac{C_{e}}{q_{m}}+\frac{1}{q_{m}b},$$

Freundlich equation:

(5) $$lnq_{e}=lnK_{f}+\frac{1}{n}C_{e},$$

Tempkin equation:

(6) $$q_{e}=\frac{RT}{b_{T}}\left(lnA\right)+\frac{RT}{b_{T}}\left(lnC_{e}\right)$$

where *q_e_* is the equilibrium adsorption capacity of the adsorbent in mg/g, *C_e_* is the equilibrium Ni(II) concentration in mg/L, *q_m_* is the monolayer adsorption capacity (mg/g), and the other parameters are different isotherm constants, which can be determined from the non-linear regression of the adsorption experimental data.

The adsorption isotherms are shown in Figure S3 and estimated isotherm parameters, as well as correlation coefficients, are listed in Table 4. Compared to Langmuir and Tempkin isotherm models, the higher *R^2^* (*R^2^* > 0.96) validated that the Freundlich isotherm model could describe the adsorption experimental data well. The surface properties and affinity of an adsorbent could be described by the constants of the Freundlich isotherm model [41]. In the Freundlich isotherm model, *K_f_* is the Freundlich constant, representing adsorption capacity, and *n* is the temperature dependent constant. When 1 < *n* < 2, the adsorption is easy to carry out, while when *n* < 0.5, the adsorption is difficult to proceed [36,42]. It can be seen from Table 4 that *n* of all samples were close to 1, indicating that the Ni(II) adsorption was relatively easy [43].

**Figure 8:**
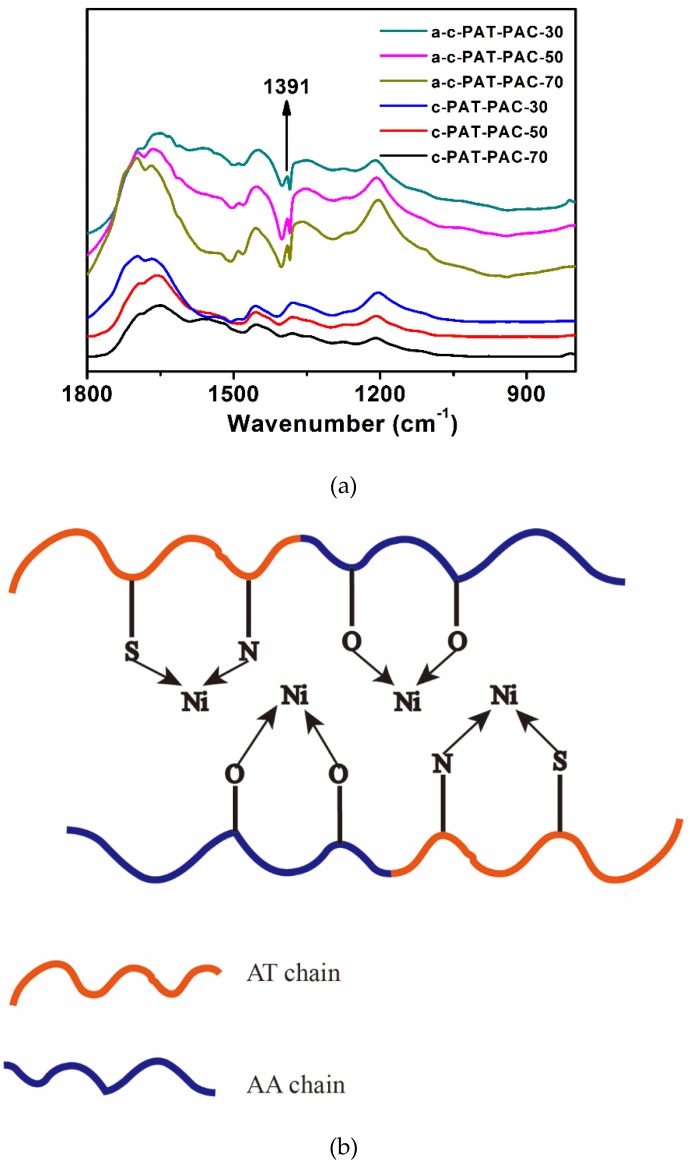
(**a**) FTIR of the chemical structure changes after Ni(II) adsorption within the spectral ranges of 1800–800 cm^−1^ and (**b**) schematic representation of the chelation cross-link reaction between PAT–PAC adsorbents and Ni(II). AT is allylthiourea, AA is acrylic acid.

In order to clarify the adsorption mechanism of Ni(II), the FTIR spectra of the as-prepared adsorbent before and after the adsorption were collected and compared. As shown in Figure 8a, a new peak emerged at 1391 cm^−1^ after the adsorption of the Ni(II) ions, suggesting that there were mutual interactions between the Ni(II) ions and the adsorbents. It was anticipated that the Ni(II) ions might interact with electron donating groups such as C=O, C=N, and C=S in the adsorbent matrix [8,12]. However, our results could not find the shift of the characteristic bands such as C=O and C=S reported in the previous references [12].

Based on FTIR spectra in Figure 8a, schematic representation of chelation cross-link reaction between adsorbents and metal ions is shown in Figure 8b. The PAT–PAC adsorbents containing O, N, and S atom, could be chelated to metal ions, showing a high selectivity for metal ion adsorption. Furthermore, our studies showed that the introduction of N, S, and other atoms to the framework could effectively increase the interaction sites with metal ions, thereby increasing the adsorption capacity [44]. Therefore, all samples had a high Ni(II) adsorption more than 500 mg/L.

### 2.3. Organic Solvent and Dye Adsorption

**Figure 9:**
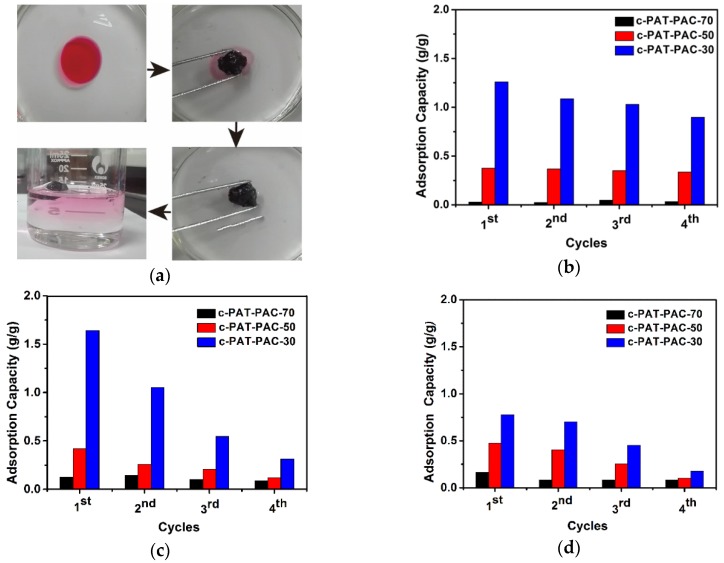
(**a**) Removal of spilled oil film (paraffin oil) from water by carbon monoliths and adsorption capacities of carbon monoliths for (**b**) acetone, (**c**) paraffin oil, and (**d**) soybean oil.

The as-fabricated carbon monoliths were also used to remove organic solvents. As can be seen from Figure 9a, our carbon monoliths could absorb the paraffin oil (dyed by red oil O) [16] on the water surface selectively, and the whole adsorption only lasted for 10 s. After that, the above carbon material was soaked in acetone [45,46] for desorption. For the same organic solvent (Figure 9b–d), the adsorption amount of c-PAT–PAC-30 reached maximum first, followed by c-PAT–PAC-50, and c-PAT–PAC-70, revealing the organic solvent adsorption increased with the increasing AT. The best organic solvent adsorption capacity of c-PAT–PAC-30 was due to the porous structure with larger size and sufficient contact between porous carbon material skeleton and organic solvents [29]. As for acetone (Figure 9b), c-PAT–PAC-30 had the highest adsorption of 1.26 g/g, followed by c-PAT–PAC-50 (0.38 g/g), and c-PAT–PAC-70 (0.05 g/g). Except for c-PAT–PAC-30 holding acetone adsorption of 71.23% compared with the first time, c-PAT–PAC-50, and c-PAT–PAC-70 kept almost constant after 4 cycles. It was clear that the paraffin oil adsorption of c-PAT–PAC-50 reached a peak of 1.64 g/g while c-PAT–PAC-50 was 0.42 g/g and c-PAT–PAC-70 was 0.14 g/g. After four adsorption–desorption cycles, c-PAT–PAC-30 only kept adsorption of 19.14% than before and that of c-PAT–PAC-30 was 28.38% but c-PAT–PAC-30 held 69.21% (Figure 9c). In addition, it is worthy to point out that c-PAT–PAC-30 had a lower soybean oil adsorption of 0.78 g/g, followed by c-PAT–PAC-50 (0.47 g/g), and c-PAT–PAC-70 (0.16 g/g). However, after 4 cycles, c-PAT–PAC-30, c-PAT–PAC-50, and c-PAT–PAC-70 retained adsorption of 22.69%, 21.59%, and 49.72%, respectively, compared with the first adsorption (Figure 9d). Our findings show that the carbon monoliths may be an ideal candidate for the adsorption of organic solvents from water owing to their surface hydrophobicity, high porosity, and oleophilicity.

**Figure 10:**
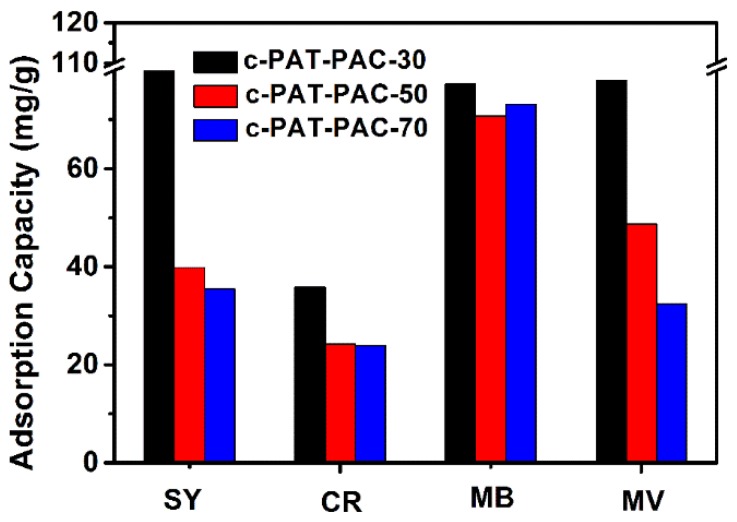
Dye adsorption of carbon monoliths. SY is sunset yellow, CR is congo red, MB is methylene blue, and MV is methyl violet.

Through spectrophotometry, the dye adsorption of dry hydrogels and carbon monoliths was obtained. Dry hydrogels had the greatest adsorption capacity for methyl violet (MV) (41 mg/g), followed by methylene blue MB (39 mg/g), and sunset yellow (SY) (3 mg/g) while there was no adsorption for congo red (CR) (Figure S4). In other words, PAT–PAC-n hydrogels behaved better with cationic dye adsorption than anionic dye adsorption. The reason is that PAT–PAC-n hydrogels contained –COOH and –NH_2_, and the cationic dyes had an electrostatic interaction with these anions, causing the dyes to permeate into the hydrogel network to show better adsorption capacity [47,48]. PAT–PAC-30 had the best ability to adsorb dyes, showing that the dye adsorption capacity of dry hydrogels was positively correlated with the AT content. After carbonization, all the adsorption capacities of SY, CR, MB, and MV were enhanced significantly (Figure 10). Particularly, both the adsorption capacities of anionic dyes (SY and CR) were higher than 24 mg/g. Compared to dry hydrogels, the adsorption capacity of c-PAT–PAC-n for MB increased more than twice. Based on the FTIR, XPS, and SEM analysis above, this enhanced adsorption may have been due to the π–π electron donor acceptor interactions originating from the benzene rings in both dye molecules and the conjugated C=C network in carbon materials as well as the abundant pores in carbon materials [49,50]. Compared with the previous reports in Table 5, c-PAT–PAC-30 was a more effective adsorbent for methylene blue removal. 

## 3. Materials and Methods

### 3.1. Reagents

Acrylic acid (AA), allylthiourea (AT), azodiisobutyronitrile (AIBN), and ethylene glycol dimethacrylate (EGDMA) were purchased from Shanghai Macklin Biochemical Co., Ltd. (Shanghai, China). Isopropyl alcohol (IPA) and paraffin liquid (Chemically Pure) were provided by Guangdong Guanghua Technology Co., Ltd. (Shantou, China). Being consistent with copper acetate monohydrate (C_4_H_6_CuO_4_·H_2_O) and nickel chloride hexahydrate (NiCl_2_·6H_2_O), with addition of 2,9-dimethyl-1,10-phenanthroline and dimethylglyoxime, methyl violet (MV) was obtained from Shanghai Macklin Biochemical Co., Ltd. (Shanghai, China). Sunset yellow (SY) and congo red (CR) were received from Shanghai Yuan Ye Biological Technology Co., Ltd. (Shanghai, China) while methylene blue trihydrate (MB) was purchased from West Long Chemical Co., Ltd. (Shantou, China). All the chemicals used were of analytical grade and used without any purification. Distilled water and absolute alcohol were used throughout unless stated otherwise.

### 3.2. Hydrogel Preparations

A series of poly (allylthiourea-co-acrylic acid) hydrogels were synthesized in isopropanol using ethylene dimethacrylate (EGDMA) and azodiisobutyronitrile (AIBN) as crosslinking reagent and initiator, respectively (Table 6). The mixtures were then allowed to react at 70 °C for 4 h with the protection of nitrogen gas. After that, the as-synthesized hydrogels were cut into small disks and soaked in 200 mL 50 wt.% ethanol solution twice (5 min per time) at room temperature to remove unreacted monomer, crosslinking agent, and initiator. After that, the hydrogels were dried in a vacuum oven at 80 °C for 48 h. The as-synthesized block hydrogels were named as PAT–PAC-*n*, where n represents the molar percentage of acryl acid monomer used (Table 6). For comparison, pure poly allylthiourea and poly acryl acid hydrogels were also synthesized using the same procedures and denoted as PAT and PAC.

### 3.3. Characterizations

Swelling ratios (SR) were determined using a gravimetric method. Hydrogel disks (around 1.20 g) were immersed in 100 mL Na2HPO4–NaH2PO4 buffers with pH 4.00, 7.00, and 9.00, respectively. The swelling hydrogels were then weighted at predetermined times until the weights reached swelling balance. The hydrogel-swelling ratios (SR) were calculated under the following formula:

(7) $$SR\left(\%\right)=\frac{m_{t}-m_{d}}{m_{d}}\times 100\%,$$

where *m_t_* and *m_d_* are the weight of the swollen and original dried hydrogels, respectively.

Fourier transform infrared spectroscopy (FTIR) measurements were carried out to determine the functional groups using the Spectrum 100 (PerkinElmer, Waltham, MA, USA). The spectra were recorded using 32 scans and 4 cm^−1^ resolution ranging from 400–4000 cm^−1^.

Thermal analysis was carried out in nitrogen gas using a TG/DTA 6300 thermal analyzer (PerkinElmer, Waltham, MA, USA). Then, 4–6 mg hydrogel was heated to 600 °C at a heating ratio of 10 °C/min. The weight loss percentage was recorded to determine the thermal stability. Based on thermal stability, the carbonization was performed using a vacuum/atmosphere tube furnace (SK-G06123K. Zhonghuan, Tianjin, China) under nitrogen gas protection. All hydrogels were heated to 300 °C at a heating rate of 5 °C/min and then kept isothermally for 30 min. After that, all hydrogels were cooled down at a cooling rate of 5 °C/min. The carbon monoliths were named as c-PAT–PAC-n, where n represents the molar percentage of acryl acid monomer used as well. The carbonization yield was defined as:

(8) $$Y\left(\%\right)=\frac{m_{c}}{m_{g}\;}\times 100\%,$$

where *m_c_* and *m_g_* are the weight of the samples after carbonization and before carbonization, respectively.

X-ray photoelectron spectroscopic (XPS) spectra were used to detect chemical structures using a K-Alpha X-ray photoelectron spectrometer (Thermo Fisher Scientific, Waltham, MA, USA). Survey spectra were obtained at pass energy of 100 eV to obtain the composition in terms of elemental C, O, N, and S.

Morphological changes were observed using SEM model S-4800 (Hitachi, Tokyo, Japan) at an accelerating voltage at 3 kV. All cross-sections were coated with gold before investigation.

### 3.4. Heavy Metal Ion Adsorption

Ni(II) was selected as the model ion for adsorption analysis. An amount of 0.200 g of carbon monoliths was added into a 100mL conical flask containing 40 mL Ni(II) solution (600 mg/L) for consecutive 3 days at 30 °C to reach equilibrium. The concentration change of Ni(II) in the solution was then measured using dimethylglyoxime (GB/T 223.23-2008) as complexing agent by an UV–visible spectrophotometer (UV2800S, Yaochunhengyu) [12]. The mechanism of GB/T 223.23-2008 is summarized as follows. Ni(II) ions and dimethylglyoxime can form a red chelate in the presence of oxidizing agent ammonium persulfate under alkaline solution. The as-obtained chelate can be analyzed spectrophotometrically at 469 nm. The adsorption capacity for Ni(II) was then calculated using the equation (9). In addition, the adsorbed adsorbents were named as a-c-PAT–PAC-*n*, and the FTIR spectra of these adsorbed adsorbents were collected to clarify the adsorption mechanism.

(9) $$Q_{e}=\frac{\left(c_{o}-c_{e}\right)\times V}{m},$$

where *Q_e_* is the adsorption capacity (mg/g), *c_o_* and *c_e_* are the initial and equilibrium concentration (mg/L), *V* is the volume (L), and *m* is the adsorbent weight (g).

Furthermore, the adsorption kinetics was investigated in a series of plastic reaction cups containing about 0.200 g adsorbent and 400 mL 600 mg/L Ni(II). Furthermore, the adsorption isotherm was obtained in detail by agitating adsorbent in the Ni(II) ion solutions with concentrations ranging from 200 to 1000 mg/L. Both the kinetics and the isotherm were done for a given time equal to the respective equilibrium time. The kinetics and isotherm were calculated using equation (9).

In order to explore the adsorbent-reusability, desorption studies were performed by dispersing these Ni(II)-loaded PAT–PAC adsorbents into 40 mL EDTA solution (0.01 mol/L) and agitating at 30 °C for 30 min.

### 3.5. Organic Solvent and Dye Adsorption

First, 0.200 g carbon monoliths were poured into 40 mL organic solvent solutions, including acetone, paraffin oil, and soybean oil. After 3 days continuous adsorption, the organic solvent-loaded carbon monoliths were separated by direct filtration and volatilized at room temperature for a period of time before being dried in a blast drying oven at 80 °C for one day; then the weight was recorded. To determine the reusability, a consecutive adsorption–desorption cycle was repeated for 4 times by using acetone as eluent. Most importantly, the adsorption capacity was calculated according to the following equation:

(10) $$Q_{e}=\frac{m_{e}}{m_{0}},$$

where *Q_e_* is the adsorption capacity (g/g), *m_0_* and *m_e_* are the initial and equilibrium weight (g), respectively.

The removal of dyes like sunset yellow (SY), congo red (CR), methylene blue (MB), and methyl violet (MV) was investigated on an UV–visible spectrophotometer (UV2800S, Yaochunhengyu). First of all, the carbon monolith (0.200 g) was added into dye solution (40 mL) with a concentration of 200 mg/L and agitated for 3 days. Then according to the standard curve, the concentration of residual dye in the solution was determined at 481 nm (SY), 499 nm (CR), 664 nm (MB), and 582 nm (MV), respectively. The amount of adsorption was consistent with the Equation (9).

## 4. Conclusions

Poly (allylthiourea-co-acrylic acid) hydrogels were successfully synthesized and carbonized. The as-obtained carbon monoliths with abundant oxygen, nitrogen, and sulfur atoms doped in-situ, show good removal capacity for Ni(II) ions, organic solvents and dyes. The adsorption equilibrium of Ni(II) ion can be quickly realized within two hours, and it can be well described by pseudo-second-order and Freundlich models under experimental conditions. Particularly, it is found that the PAT–PAC carbon monoliths have good re-adsorption ability for Ni (II) and organic solvents. Thus, poly (allylthiourea-co-acrylic acid) derived carbon materials will show excellent prospects in industrial effluents.

## Figures and Tables

**Table 1:** XPS analysis of the PAT–PAC and c-PAT–PAC.

Samples	C (at.%)	O (at.%)	S (at.%)	N (at.%)
PAT–PAC-70	65.98	24.04	2.98	7.0
PAT–PAC-30	66.66	13.46	6.16	13.72
c-PAT–PAC-70	74.91	13.78	1.23	10.07
c-PAT–PAC-30	75.02	8.26	2.10	14.63

**Table 2:** The comparison of adsorbents toward Ni(II) adsorption reported from the literature.

Adsorbents	Adsorption Capability (mg/g)	Reference
c-PAT–PAC-30	557 (pH = 4.0, 30 °C)	This work
δ-MnO_2_/polymer millimeter-sized bead	29 (pH = 4.0, 30 °C)	[31]
chitosan/sporopollenin microcapsules	13 (pH = 4.0, 25 °C)	[32]
grafted hydrazinyl amine magnetite–chitosan	82 (pH = 4.0, 25 °C)	[33]

**Table 3:** Pseudo first-order model and pseudo second-order model constants and correlation coefficients for Ni(II) adsorption onto PAT–PAC adsorbent.

Adsorbent	Pseudo First-Order Model		Pseudo Second-Order Model		
	*K_1_* (min^−1^)	*R^2^*	*K_2_* (g mg^−1^ min^−1^)	*h* (mg/g min)	*R^2^*
c-PAT–PAC-30	0.0388	0.6002	0.0004	416.7	0.9983
c-PAT–PAC-50	0.0626	0.8566	0.0003	416.7	0.9990
c-PAT–PAC-70	0.0405	0.7062	0.0005	666.7	0.9995

**Table 4:** Freundlich isotherm model and Tempkin isotherm model constants and correlation coefficients for Ni(II) adsorption onto PAT–PAC adsorbent.

Adsorbent	Langmuir Model			Freundlich Model			Tempkin Model	
	*q_m_* (mg g^−1^)	*b* (L mg^−1^)	*R^2^*	*n*	*K_f_* (L g^−1^)	*R^2^*	*A* (L mg^−1^)	*R^2^*
c-PAT–PAC-30	–2500	–6141	0.3227	0.9655	2.2648	0.9940	0.0636	0.9085
c-PAT–PAC-50	2500	6397	0.0611	1.0240	2.6735	0.9657	0.0731	0.9508
c-PAT–PAC-70	–25,000	–82,183	0.0027	0.9748	2.9988	0.9875	0.0692	0.9613

**Table 5:** The comparison of adsorbent toward methylene blue adsorption reported from the literature.

Adsorbents	Adsorption Capability (mg/g)	Reference
PAT–PAC-30	39 (pH = 5.27, 30 °C)	Present study
c-PAT–PAC-30	110 (pH = 5.27, 30 °C)	Present study
tannin-supported on cellulose microfibers	12 (pH = 5.25, 30 °C)	[51]
purified diatomite via thermo–chemical treatment	28.5 (pH = 5.25, 25 ± 2 °C)	[52]
HY zeolites (HY (16.6))	100 (pH = 5.25, 25 °C)	[53]

**Table 6:** Hydrogel compositions.

Hydrogel	AA/ mol	AT/ mol	IPA/ g	EGDMA/ mol	AIBN/ mol
PAT–PAC-70	0.07	0.03	18	0.01	0.005
PAT–PAC-50	0.05	0.05	18	0.01	0.005
PAT–PAC-30	0.03	0.07	18	0.01	0.005

## Supplementary Materials

The following are available online, Figures S1–S4.
