# Supplementary material for: Preparation of Poly (Allylthiourea-Co-Acrylic Acid) Derived Carbon Materials and Their Applications in Wastewater Treatment
Source: Molecules. 2019 Mar 8;24(5):957. doi: 10.3390/molecules24050957 (PMC6429361; doi:10.3390/molecules24050957)
Supplement: Supplementary file 1 [file molecules-24-00957-s001.pdf]

Supplementary Materials

Article

Preparation of Poly (Allylthiourea-Co-Acrylic Acid) Derived Carbon Materials and Their Applications in Wastewater Treatment

Limei Liang [1,2], Chengpeng Li [1,2], Tingting Hou [2], Zhiying Zhong [2], Dongchu Chen [1], Sidong Li [2], Zhang Hu [2], Haihua Yang [2] and Xiufang Ye [1,]*

[1] School of Materials Science and Energy Engineering, Foshan University, Foshan 528000, China; llmgdou2017@163.com (L.L.); lcp0802@126.com (C.L.); chen@fosu.edu.cn (D.C.)

[2] Faculty of Chemistry and Environmental Science, Guangdong Ocean University, Zhanjiang 524088, China; htt0415@126.com (T.H.); zzy15767018762@163.com (Z.Z.); huzhcarrot@163.com (Z.H.); gdouyanghaihua@163.com (H.Y.)

* Correspondence: yexiufang2018@126.com; Tel.: +86-1372-667-8743

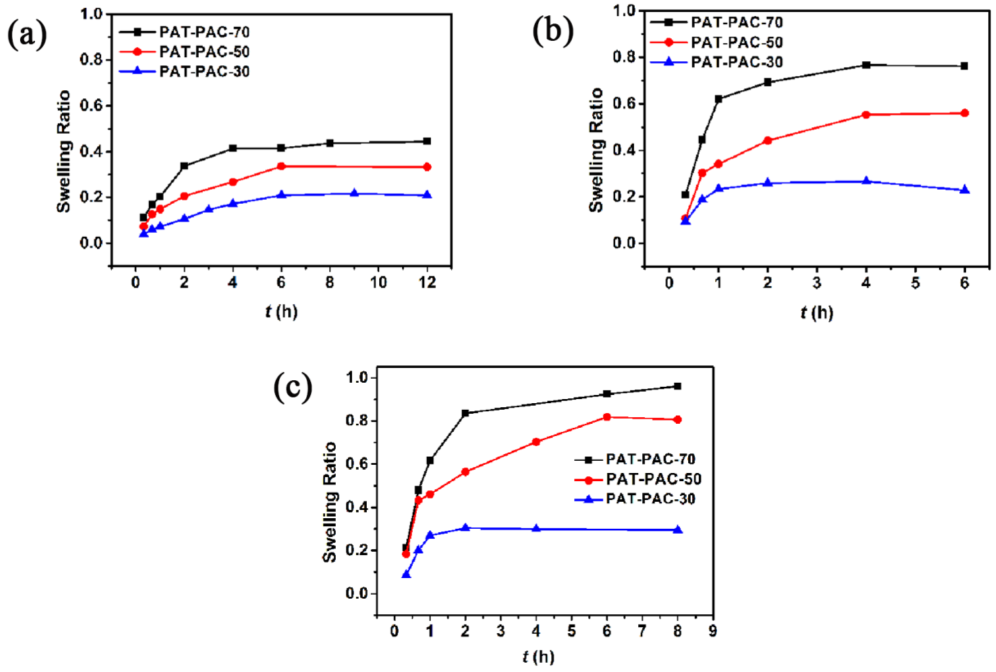

Figure S1. Swelling behaviors of PAT-PAC hydrogels at (a) pH=4.00, (b) pH=7.00 and (c) pH=9.00

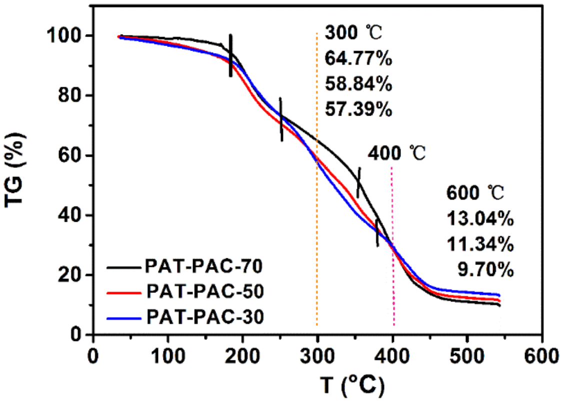

Figure S2. The thermal analysis curve of PAT-PAC hydrogels

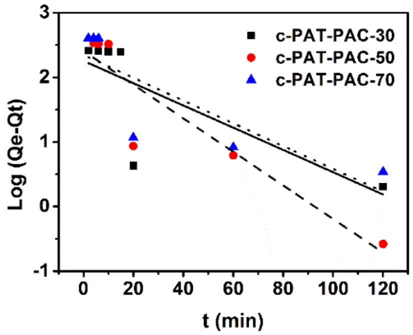

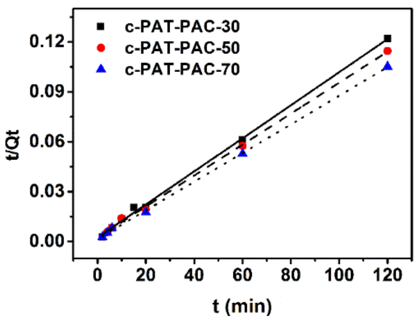

(a) **(b)**

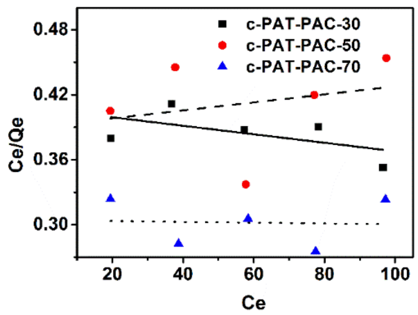

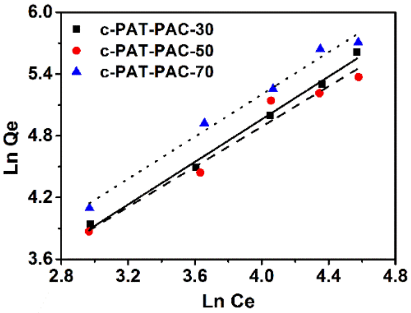

(c) **(d)**

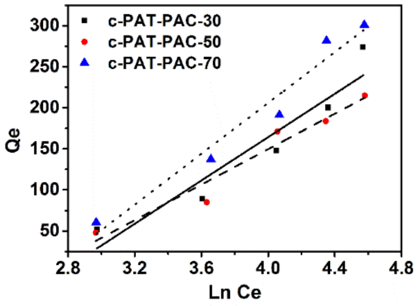

(e)

Figure S3. **(a)** Pseudo-first-order kinetics isotherms, **(b)** Pseudo-second-order kinetics isotherms, **(c)** Langmuir isotherm model, **(d)** Freundlich isotherm model and **(e)** Tempkin isotherm model for Ni(II) adsorption on carbon monoliths

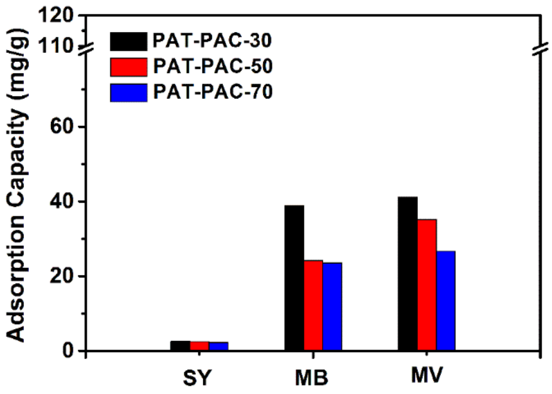

Figure S4. Dye adsorption of dry PAT-PAC hydrogels
